# Metabolic Regulation of Organic Acid Biosynthesis in *Actinobacillus succinogenes*

**DOI:** 10.3389/fbioe.2019.00216

**Published:** 2019-09-18

**Authors:** Wenming Zhang, Qiao Yang, Min Wu, Haojie Liu, Jie Zhou, Weiliang Dong, Jiangfeng Ma, Min Jiang, Fengxue Xin

**Affiliations:** ^1^State Key Laboratory of Materials-Oriented Chemical Engineering, College of Biotechnology and Pharmaceutical Engineering, Nanjing Tech University, Nanjing, China; ^2^Jiangsu National Synergetic Innovation Center for Advanced Materials, Nanjing Tech University, Nanjing, China

**Keywords:** *Actinobacillus succinogenes*, organic acid, pyruvate formate lyase 1-activating enzyme, metabolic engineering, fermentation

## Abstract

*Actinobacillus succinogenes* is one of the most promising strains for succinic acid production; however, the lack of efficient genetic tools for strain modification development hinders its further application. In this study, a markerless knockout method for *A. succinogenes* using in-frame deletion was first developed. The resulting Δ*pflA* (encode pyruvate formate lyase 1-activating protein) strain displayed distinctive organic acid synthesis capacity under different cultivation modes. Additional acetate accumulation was observed in the Δ*pflA* strain relative to that of the wild type under aerobic conditions, indicating that acetate biosynthetic pathway was activated. Importantly, pyruvate was completely converted to lactate under anaerobic fermentation. The transcription analysis and enzyme assay revealed that the expression level and specific activity of lactate dehydrogenase (LDH) were significantly increased. In addition, the mRNA expression level of *ldh* was nearly increased 85-fold compared to that of the wild-type strain during aerobic–anaerobic dual-phase fermentation, resulting in 43.05 g/L lactate. These results demonstrate that *pflA* plays an important role in the regulation of C3 flux distribution. The deletion of *pflA* leads to the improvement of acetic acid production under aerobic conditions and activates lactic acid biosynthesis under anaerobic conditions. This study will help elaborate the mechanism governing organic acid biosynthesis in *A. succinogenes*.

## Introduction

Microbial production of organic acid from renewable resources is a promising and sustainable approach due to its ecological and economical advantages (Kamzolova et al., 2014). For example, succinic acid has been identified as one of the top 12 chemical building blocks according to the US Department of Energy, which can be synthesized through microbial fermentation (Mckinlay et al., 2007b; Zhang et al., 2018b). *Actinobacillus succinogenes*, a facultative anaerobe and natural succinate producer, is one of the most promising strains for succinic acid production due to its wide carbon utilization spectrum and robustness to environment (Mckinlay et al., 2005; Pateraki et al., 2016). Various low-cost feedstocks have been used for succinate production by using *A. succinogenes*, including corncob (Zhong et al., 2015), corn stover (Borges and Pereira, 2011), sugarcane bagasse hemicellulose hydrolysate (Li et al., 2011), etc. It should be noticed that other organic acids, such as pyruvic and lactic acids, were also accumulated under different cultivation modes, taking advantage of its unique incomplete TCA cycle. For instance, *A. succinogenes* NJ113 has been reported to be a potential industrial application for the bioproduction of pyruvic acid under microaerobic fermentation condition, and dissolved oxygen environment was found to have a vital role in promoting pyruvic acid production (Wang et al., 2016). Initial oxygen aeration was also necessary to enhance lactic acid biosynthetic capacity for *A. succinogenes* 130Z^T^ in subsequent anaerobic cultivation (Li et al., 2010). Conversely, physiological changes during aerobic growth of engineered *E. coli* strain could significantly affect succinate production in the subsequent anaerobic phase (Vemuri et al., 2002).

Currently, metabolic engineering strategy has attracted increasing attention to develop industrially competitive microorganisms, such as *Mannheimia succiniciproducens, Escherichia coli, Corynebacterium glutamicum, Clostridium acetobutylicum, Saccharomyces cerevisiae*, and *Yarrowia lipolytica* (Li et al., 2016; Ahn et al., 2017; Chung et al., 2017; Cui et al., 2017; Franco-Duarte et al., 2017; Xue et al., 2017). To improve succinic acid production, some cases regarding genetic modification of *A. succinogenes* have been reported, including enhancement of pathways involved in succinic acid synthesis and deletion of pathways involved in byproduct accumulation (Joshi et al., 2014; Guarnieri et al., 2017). For example, overexpression of the key enzymes (such as malate dehydrogenase) in the reductive branch of the TCA cycle enhanced flux to succinic acid, resulting in titer, rate, and yield enhancements (Guarnieri et al., 2017). A single-knockout mutant of the pyruvate formate lyase (PFL, encoded by *pflB*) was obtained using natural transformation method employing a positive selection marker (isocitrate dehydrogenase), which will effectively eliminate formate biosynthesis (Joshi et al., 2014). Thereafter, a series of single-knockout mutants and double-knockout mutants were constructed using electroporation employing antibiotic resistance marker to drive carbon flux into succinic acid (Guarnieri et al., 2017). However, it should be noticed that delayed bacterial growth, sugar utilization, and succinic acid biosynthesis were observed in knockout mutants compared with the wild strain, though formation of by-products including acetate and formate were effectively reduced. Thus, the strategy to improve the production of succinic acid through the removal of competitive pathways in *A. succinogenes* was still in vain.

Compared to metabolic gene knockouts, change of corresponding protein activity might be better to improve strain's growth performance. For instance, there is an activation system composed of activating factor and coenzyme under anaerobic conditions, which can convert the inactive PFL pro-enzyme into active PFL by amino acid free radical activation (Rodel et al., 1988). Pyruvate formate lyase 1-activating protein (encoded by *pflA*) is usually an activator to *pflB*. It was reported that deletion of *pflA* can increase succinic acid production from glycerol in *E. coli* under anaerobic conditions (Mienda et al., 2018). Protein homology analysis method showed that *pflA* can activate *pflB* under anaerobic conditions in *A. succinogenes* (Zhang et al., 2018a). These data provide a novel gene knockout target for increasing succinate production in *A. succinogenes*.

In this study, genetic tools for markerless gene knockout by in-frame deletion was first developed. The metabolic regulation of organic acid biosynthetic in *A. succinogenes* Δ*pflA* strain under different fermentation strategies was also investigated. To elucidate the mechanism underlying the increased different organic acids production, the transcriptional level of key genes involved in metabolic pathway was evaluated. In this work, the effect of the deletion of *pflA* on organic acids production in *A. succinogenes* was studied. Additionally, the engineered strain represents a promising role for acetate or lactate production.

## Materials and Methods

### Strains and Media

*Actinobacillus succinogenes* 130Z (ATCC 55618) was obtained from the American Type Culture Collection. Wild-type and engineered strains were cultivated in sterile seed medium YC (containing the following: 5 g/L yeast extract, 9.6 g/L NaH_2_PO_4_·2H_2_O, 15.5 g/L K_2_HPO_4_·3H_2_O, 10 g/L NaHCO_3_, 10 g/L glucose, 1 g/L NaCl, and 2.5 g/L corn steep liquor) at 37°C and 200 rpm. *E. coli* SM10 λ*pir*, provided by Professor Lianrong Wang (Wuhan University), was used as the donor strain to allow efficient replication of the suicide plasmid and grown in Luria-Bertani (LB) medium. The fermentation medium consisted of 10 g/L yeast extract, 1.36 g/L NaAc, 0.3 g/L Na_2_HPO_4_·12H_2_O, 1.6 g/L Na_2_HPO_4_·2H_2_O, 3 g/L K_2_HPO_4_, 1 g/L NaCl, 0.2 g/L MgCl_2_·6H_2_O, 7.5 g/L corn steep liquor, and 0.2 g/L CaCl_2_. Glucose was added after sterilization at a concentration of 30–50 g/L for fermentation.

### DNA Manipulation and Conjugation

To construct a knockout cassette, the 1.5-kb up- and down-stream regions of *pflA* gene were individually amplified by PCRs from genomic DNA of *A. succinogenes* using primers listed in Supplementary Table 1. Two purified fragments were ligated to a shuttle suicide plasmid pJC4 using One Step Cloning Kit (Vazyme, China), thereafter naturally transformed into *E. coli* SM10 λ*pir*.

Recipient strain *A. succinogenes* and donor bacteria were grown in media until exponential phase. Cells were mixed at an equal ratio. Then, mixture cultures were harvested by centrifugation (4,500 × *g*, 3 min) and washed three times with LB medium at room temperature. Pellets were resuspended in 50 μl of LB medium and spotted onto an LB plate. During anaerobic cultivation at 37°C, the shuttle suicide plasmid might enter the recipient strain from the donor bacteria. After 16 h of conjugation, cells were washed with LB medium and plated on the YC medium. For the selection of the first crossing-over and elimination of the donor strain, 34 μg/ml of chloramphenicol was supplemented. Clones were checked by PCR analysis (with primers F_0_ and down-rev, up-fwd and R_0_). Subsequently, positive colony was plated on YC agar plates with 15% sucrose, which is a counter selectable marker to promote the second crossing-over. The plasmid could be excited from the chromosome, generating wild-type strain or deletion mutant. Clones were checked with primers *pflA*-F_0_ and *pflA*-R_0_ by colony PCR.

### Fermentation in Seal Bottles and Bioreactors

A 6% seed inoculum from overnight YC culture was added to 100-ml sealed bottles containing 30 ml of fermentation medium supplement with 30 g/L glucose and 16 g/L magnesium carbonate hydroxide to maintain the pH at 6.8 and the fermentation was carried out at 200 rpm and 37°C. The anaerobic condition was achieved by sparging CO_2_ for 2 min (Dessie et al., 2018).

Bioreactor fermentations were performed in a 5-L fermentor (Bioflo 110, USA) containing 2 L of fermentation medium. The temperature was controlled at 37°C and the agitation at 300 rpm. The pH was maintained at 6.8 by supplementing 20% Na_2_CO_3_. During aerobic fermentation conditions, air was sparged at 1 vvm. While anaerobic conditions were created by sparging with CO_2_ at a flow rate of 0.2 L/min. When the glucose concentration was lower than 10 g/L, 600 g/L glucose was fed into the fermentation medium until the final concentration was 50 g/L.

### Enzymatic Assays

Cultures were harvested by centrifugation (4°C, 10,000 × *g*, 10 min) and washed twice with 50 mM Tris-HCl buffer (pH at 7.0). Pellets were resuspended in 100 mM Tris–HCl buffer (pH at 7.0) containing 2 mM DTT and 0.1 mM EDTA. Cell disruption by sonication was followed *via* centrifugation for 10 min at 10,000 × *g* at 4°C. The supernatant was used for all enzyme assays. The protein concentration was determined by the method of Bradford (1976).

The enzyme activity of pyruvate formate lyase was measured as described previously (Melchiorsen et al., 2002). The lactate dehydrogenase (LDH) assay was performed based on Vanderwerf et al. (1997). One unit (U) was defined as the amount of enzyme catalyzing the conversion of 1 μmol of substrate per minute into specific products. Specific activity was defined as units per milligram of protein (Li et al., 2010).

### Quantitative RT-PCR

Cultures grown under different cultivation conditions were subject to RNA extraction during 8 h in aerobic/anaerobic or 20 h in dual-phase fermentation. Takara RNAiso Plus (Takara) was used to extract total RNA. After eliminating genomic DNA by RNase-free DNase I (Takara), RNA was converted into cDNA using a cDNA synthesis kit (Takara).

SYBRs Premix Ex TaqTM kit purchased from Takara was used for RT-PCR. All experiments were performed in biological triplicates in 96-well plates using the ABI 7500 Real-Time PCR system (Applied Biosystems). The 16S rRNA gene was used to standardize the mRNA levels. Primer sequences for qRT-PCR are indicated in Supplementary Table 1. The mRNA level of the target genes was calculated *via* the 2^−ΔΔ*CT*^ method (Livak and Schmittgen, 2001).

### Analytical Methods

Cell growth was determined by OD_600_ in an AOE INSTRUMENTS-UV1800 spectrophotometer. Organic acids were measured by high-performance liquid chromatography (UitiMate 3000 HPLC system, Dionex, USA) equipped with an ion exchange column (Bio Rad Aminex HPX-87H column, USA) at 55°C, using a mobile phase of 5 mM H_2_SO_4_ at a flow rate of 0.6 ml/min and a UVD 170U ultraviolet detector at 215 nm for detection, as described previously (Zhang et al., 2018c). Glucose concentration in the fermentation broth was detected by a chronoamperometry method. In this detection process, a calibration line of current response vs. glucose concentration was first fitted by the continuous additions of a standard glucose concentration into a buffer solution. Then, the target sample was added to calculate its glucose concentration by using the calibration line (Jiang et al., 2016; Yang et al., 2017).

## Results and Discussion

### A Markerless Knockout Method for *A. succinogenes* by In-Frame Deletion

**Figure 1:**
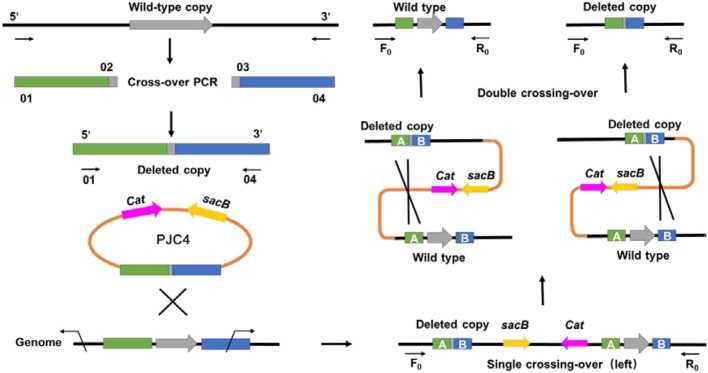
General diagram representing an alternative method for markerless gene deletion by allelic exchange in *Actinobacillus succinogenes*.

Different from previous transformation methods, which adopted the positive selection marker with natural transformation or antibiotic resistance marker with electroporation (Joshi et al., 2014; Guarnieri et al., 2017), a markerless knockout method for *A. succinogenes* using conjugation was first developed. To create the gene deletion method in *A. succinogenes*, a shuttle suicide vector (pJC4) was employed, which carried *R6K ori* (the replication functional in *E. coli*), *cat* gene conferring chloramphenicol resistance, and *sacB* gene from *Bacillus subtilis* (the sucrose-sensitivity system). The replacement cassette contains two 1.5-kb regions flanking the target gene on the chromosome of *A. succinogenes*. After single crossing-over screened by chloramphenicol resistance and second crossing-over by sucrose counter selection, a markerless knockout mutant was obtained (Figure 1). Although markerless knockout method has been reported, the residual “scar” still exists in the chromosome at the site of the deletion, such as Flp sites, which will lead to large sections of the genome rather than two FRT sequences flanking the marker incorrect deletion when multiple genes have been consecutively deleted from one strain (Joshi et al., 2014). The mutants generated through in-frame deletion method in this study solved this problem efficiently and achieved real markerless knockout, providing a research basis for the metabolic engineering of *A. succinogenes*. Based on this method, pyruvate formate lyase 1-activating protein encoded by *pflA* in *A. succinogenes* 130Z was successfully knocked out and mutant was generated markerless.

### Redistribution of Metabolic Products by Mutant With *pflA* Deletion

Generally, metabolic flux in *A. succinogenes* will be split into the following two branches under anaerobic conditions: (i) C3 pathways for formate, acetate, ethanol, and lactate production, and (ii) C4 pathway for succinate production. As known, the insufficient supply of reducing power is one main obstacle to achieve high succinic acid yield under anaerobic conditions, as succinic acid yield from glucose or other carbon sources strongly depends on ATP and NADH levels, which were mainly produced by C3 pathways (Singh et al., 2011; Zhang et al., 2018c). However, the accumulation of by-products synthesized through C3 pathways will not only reduce the substrate utilization but also cause difficulties in the separation and purification steps. It was found that formate could be significantly reduced when PFLB catalyzing pyruvate into acetyl coenzyme A and formate was knocked out. However, the cell growth rate was also significantly decreased in Δ*pflB* strain. Succinate yield was still similar to that of wild-type strain. To relieve the negative effect by *pflB* knockout, pyruvate formate lyase 1-activating protein (*pflA*) was further knocked out.

**Figure 2:**
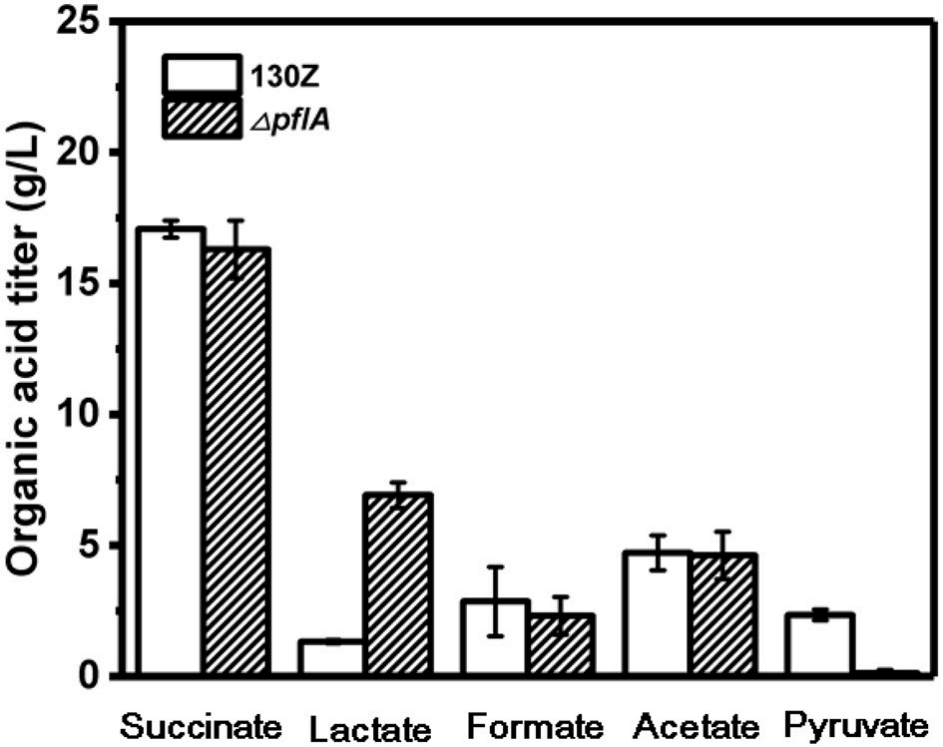
Comparison of different organic acids production between wild-type *A. succinogenes* 130Z and Δ*pflA* strains.

Wild-type and Δ*pflA* strain were cultivated in sealed bottles containing 30 g/L glucose. The titers of succinate, formate, and acetate in the Δ*pflA* strain were 15.78, 4.53, and 8.23 g/L, respectively, which were approximately equal to those of the wild-type strain (Figure 2). Results demonstrated that the absence of activating protein (PFLA) did not affect the expression of pyruvate formate lyase, and succinic acid production was not increased significantly. Interestingly, pyruvate, and lactate production were significantly changed. Pyruvate accumulation was observed in both wild-type and Δ*pflB* strains (Guarnieri et al., 2017), while traces of pyruvate accumulation (<1 g/L) were observed in the Δ*pflA* strain, indicating that pyruvic acid was completely assimilated. Surprisingly, the Δ*pflA* strain displayed a significantly increased lactate accumulation capacity (6.92 g/L) with 5.77-fold improvement, which coincided with pyruvate assimilation (Figure 2). Conversely, no lactic acid accumulation was observed in the wild-type or Δ*pflB* strains.

**Figure 3:**
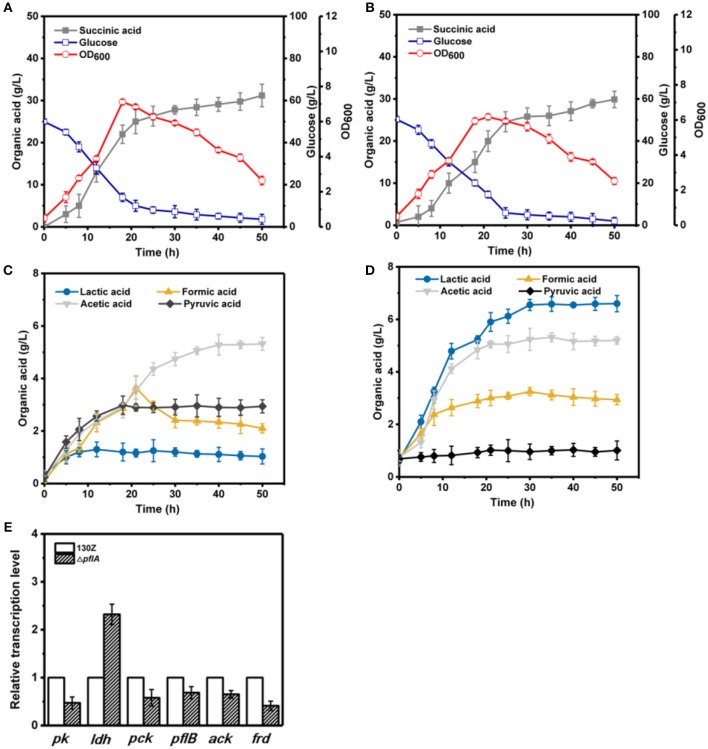
Evaluation of different fermentation and metabolic parameters in wild-type *A. succinogenes* 130Z **(A,C)** compared to those of the Δ*pflA* strain **(B,D)** under anaerobic conditions. The bar graph **(E)** represents the expression levels of genes related to organic acid biosynthesis pathway.

To further investigate the effect of *pflA* deletion on cell growth and metabolite profiles, batch fermentation of wild-type and Δ*pflA* strains was carried out under anaerobic conditions in a 5-L fermentor with 50 g/L of glucose. As illustrated in Figure 3A, 40 g/L of glucose was consumed after 18 h of fermentation by the wild-type strain, and the maximal OD_600_ reached 7.07. However, cell growth and glucose consumption rates were slightly decreased by the Δ*pflA* strain with a maximal OD_600_ of 6.07 at 21 h. Complete glucose consumption was ultimately achieved after 50 h of cultivation (Figure 3B). Delayed glucose consumption and cell growth coincided well with delayed succinic acid production relative to that of the 130Z strain. 30.25 g/L succinic acid with a yield of 0.62 g/g was achieved, which was decreased by 10% when compared to that of control strain. The strain Δ*pflA* showed similar acetic and formic acid accumulation profiles to that of wild type; acetic and formic production reached 5.32 and 2.26 g/L, respectively (Figure 3C). Notably, negligible amount of lactate accumulation was observed in strain 130Z, which is in agreement with the previously reported wild-type strain. Conversely, more lactate accumulation (6.43 g/L) and nearly complete elimination of pyruvate was observed by using the Δ*pflA* strain (Figure 3D), indicating that lactate flux was activated after removal of *pflA*. Although lactate accumulation was also observed in the Δ*pflB* Δ*ackA* strain, pyruvate was still accumulated at early exponential phase and yet completely assimilated following 24 h of cultivation (Guarnieri et al., 2017), which is different from this study. Therefore, in addition to knockout of both *ackA* and *pflB*, deletion of *pflA* can also lead to lactic acid generation, indicating that *pflA* plays a critical role in lactic acid biosynthesis in *A. succinogenes*.

### Effect of *pflA* Knockout on the Expression of Key Genes Involved in Metabolic Pathway

To examine the effect of *pflA* knockout on the gene expression in *A. succinogenes*, the expression levels of several key genes involved in succinic acid (*pck* encoding phosphoenolpyruvate carboxykinase, *frd* encoding fumarate reductase), pyruvic acid (*pk* encoding pyruvate kinase), acetic acid (*pfl* encoding pyruvate formate lyase, *ack* encoding acetate kinase), and lactic acid formation (*ldh* encoding 6-lactate dehydrogenase) were investigated. Relative mRNA transcription levels of these genes at exponential phase for strain 130Z and Δ*pflA* were measured with RT-PCR. 16S rRNA copy number was used as internal reference. As shown in Figure 3E, knockout of *pflA* could slightly down-regulate mRNA levels of all metabolite synthesis genes except *ldh*, which coincides with the prolonged bacterial growth and product synthesis in the Δ*pflA* strain. For example, the expression level of *pk* and *frd* was nearly 0.4-fold lower than that of strain 130Z. Surprisingly, the mRNA level of *ldh* in the Δ*pflA* strain was 2.3-fold higher than that of the wild-type strain, indicating that the lactic acid biosynthesis pathway might be activated by deletion of *pflA*.

In order to further investigate the metabolic regulation mechanism, the activities of two key enzymes PFL (pyruvate formate lyase) and LDH involved in C3 branch pathways were investigated. As shown in Table 1, PFL enzyme activities were similar between the wild-type (18.25 U/mg) and Δ*pflA* strains (17.65 U/mg), which is in agreement with the fermentation results. However, LDH specific activity was significantly increased in the Δ*pflA* strain, which was 2.62-fold higher than that of the wild-type strain. The maximum activity was 30.34 U/mg, which coincides well with the mRNA level and lactate production. Taken together, these results indicated that the expression of pyruvate formate lyase in *A. succinogenes* does not depend on the activation of pyruvate formate lyase 1-activating protein, though its function notation in NCBI is to activate pyruvate formate lyase 1 under anaerobic conditions. Moreover, the absence of this protein might be beneficial for the carbon flux to lactic acid. Therefore, we speculated that the function of pyruvate formate lyase 1-activating protein might be a repressor for *ldh* gene transcription, which is different from *E. coli*.

**Table 1:** Comparison of enzyme activities involved in organic acid formation in *Actinobacillus succinogenes* 130Z and Δ*pflA* grown anaerobically.

**Strain**	**Total protein (mg/ml)**	**PFL activity (U/ml)**	**Specific activity (U/mg)**	**LDH activity (U/ml)**	**Specific activity (U/mg)**
130Z	0.25 ± 0.02	4.56 ± 0.12	18.25 ± 0.26	3.25 ± 0.08	11.61 ± 0.32
Δ*pflA*	0.29 ± 0.01	4.06 ± 0.15	17.65 ± 0.42	10.62 ± 0.27	30.34 ± 1.21

### Organic Acid Production Under Different Fermentation Strategies

*Actinobacillus succinogenes* will change cell physiology and metabolite profile under different cultivation conditions, such as aerobic and anaerobic conditions. As mentioned above, the lactic acid biosynthesis pathway was activated after deletion of *pflA* under anaerobic conditions. To further investigate the effect of *pflA* deletion on cell growth and metabolic performance under aerobic and aerobic–anaerobic dual-phase cultivation modes, the fed-batch fermentation using wild-type 130Z and Δ*pflA* strain was conducted in a 5-L fermentor.

**Figure 4:**
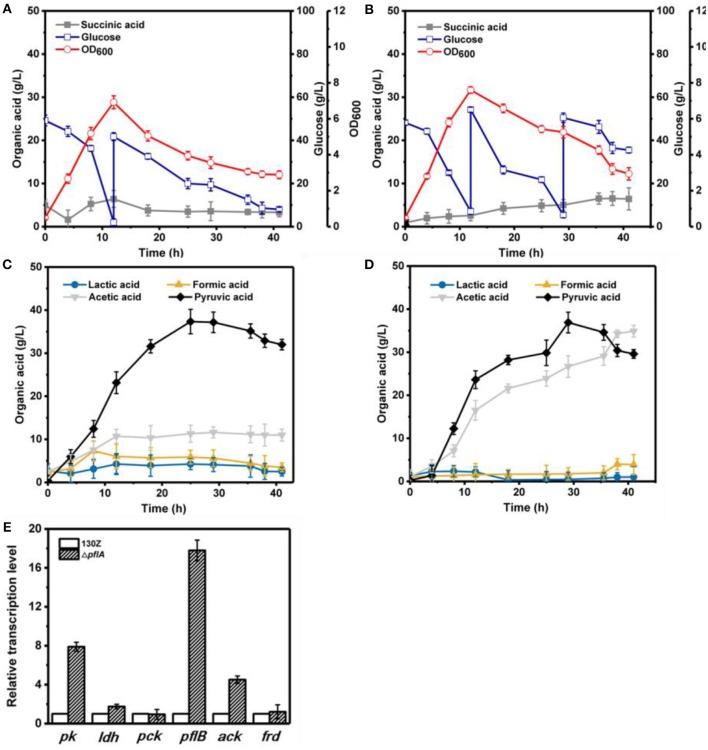
Evaluation of different fermentation and metabolic parameters in the 130Z strain **(A,C)** compared to those of the Δ*pflA* strain **(B,D)** under aerobic conditions. The bar graph **(E)** represents the expression levels of genes related to organic acid biosynthesis pathway.

Under aerobic conditions, 82 g/L of glucose was completely consumed after 41 h by strain 130Z with a maximal OD_600_ of 6.92 at 12 h (Figure 4A). Interestingly, strain Δ*pflA* displayed higher growth rate, which is obviously different from that under anaerobic conditions (Figure 4B). As a result, more glucose (105 g/L) was consumed with a maximal OD_600_ of 7.64. As a main product, pyruvic acid accumulation profiles were similar between strain Δ*pflA* and 130Z, and the highest titer reached 36.90 and 37.36 g/L, respectively. The latter is in agreement with a previous study reported under microaerobic fermentation (Wang et al., 2016). Surprisingly, massive acetic acid accumulation was also observed in the Δ*pflA* strain. The highest titer of acetic acid can reach 34.92 g/L, 13.7-fold titer enhancement (Figure 4D), indicating that acetic acid biosynthesis was enhanced and carbon flux was shifted to acetic acid after the deletion of *pflA* under aerobic conditions. As known, ATP generation is accompanying by acetic acid production (Guarnieri et al., 2017), leading to cell growth and glucose consumption improvement. Additionally, the Δ*pflA* strain displayed increased succinic acid accumulation capacity (6.43 g/L) with 48.06% enhancement. Conversely, a greater reduction in lactic and formic acid titer was observed in the Δ*pflA* strain relative to that of the wild type.

**Figure 5:**
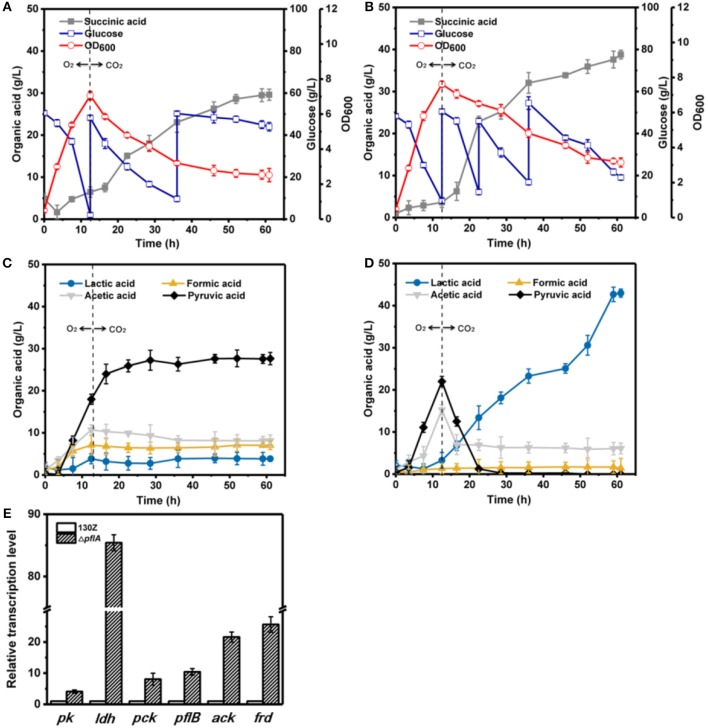
Evaluation of different fermentation and metabolic parameters in the 130Z strain **(A,C)** compared to those of the Δ*pflA* strain **(B,D)** under aerobic–anaerobic dual-phase conditions. The bar graph **(E)** represents the expression levels of genes related to organic acid biosynthesis pathway.

Under aerobic-anaerobic conditions, succinic, and pyruvic acids were main metabolic products with 29.62 and 27.64 g/L, respectively, from 96 g/L of glucose by using strain 130Z (Figure 5A). Pyruvic acid was significantly accumulated in the aerobic phase and still produced and even shifted to anaerobic condition (Figure 5C). Surprisingly, 142 g/L glucose (32.39% higher) was consumed in the Δ*pflA* strain, indicating the superior performance of bacteria growth. Final succinic acid titer (38.82 g/L) was also significantly enhanced with 23.72% improvement (Figure 5B). However, the pyruvic acid concentration was dramatically decreased in the Δ*pflA* strain when shifted to anaerobic condition. In addition, no pyruvic acid accumulation was observed at the end of fermentation with almost complete pyruvate reassimilation. As shown in Figure 5C, acetic acid production was slowly decreased under anaerobic phase with a final titer of 8.15 g/L. Moreover, lactic and formic acids production remained stable in the wild-type strain. These results showed that carbon flux distribution was shifted from C3 (pyruvic acid synthesis) toward C4 flux (succinic acid synthesis) when cultivation condition was shifted from an aerobic to an anaerobic one. By contrast, sharp accumulation of lactic acid was observed by using Δ*pflA* strain under anaerobic conditions. The maximum of lactic acid production was 43.05 g/L at 60 h of cultivation, which was a nearly 11-fold increase compared to that of the wild-type strain (Figure 5D). Conversely, the acetic acid accumulation profiles were similar between wild-type and engineered strains, and a negligible amount of formic acid was detected in the whole fermentation. These results indicated that the removal of *pflA* could significantly enhance carbon flux to lactic acid under dual-phase cultivation conditions.

### Qrt-PCR Analysis Under Different Cultivation Modes

To further elaborate the underlying mechanism responsible for organic acid synthesis, the expression levels of related genes were investigated. Previous work showed that the expression level of *ldh* in strain Δ*pflA* was much higher than that of the wild-type strain, while the expression level of other metabolite synthesis genes was slightly lower under anaerobic conditions (Figure 3). The expression levels of *pk, pfl*, and *ack* genes from the Δ*pflA* strain are significantly increased relative to that of the 130Z strain during aerobic fermentation, which coincides with acetic acid accumulation in the Δ*pflA* strain (Figures 4D,E). However, the mRNA level of all genes was significantly increased (> 4-fold) in the Δ*pflA* strain under dual-phase fermentation conditions, indicating that the strain has the superior performance of cell growth and metabolism (Figure 5E). Notably, the transcription level of *ldh* was increased nearly 85-fold, displaying a dramatically increased lactic acid accumulation capacity relative to that of the wild type. It is known that LDH is indeed encoded in its genome, while no lactate was produced in *A. succinogenes* (Mckinlay et al., 2007a). The finding in the current paper suggests that a finely tuned system might be used to lactic acid biosynthesis.

**Figure 6:**
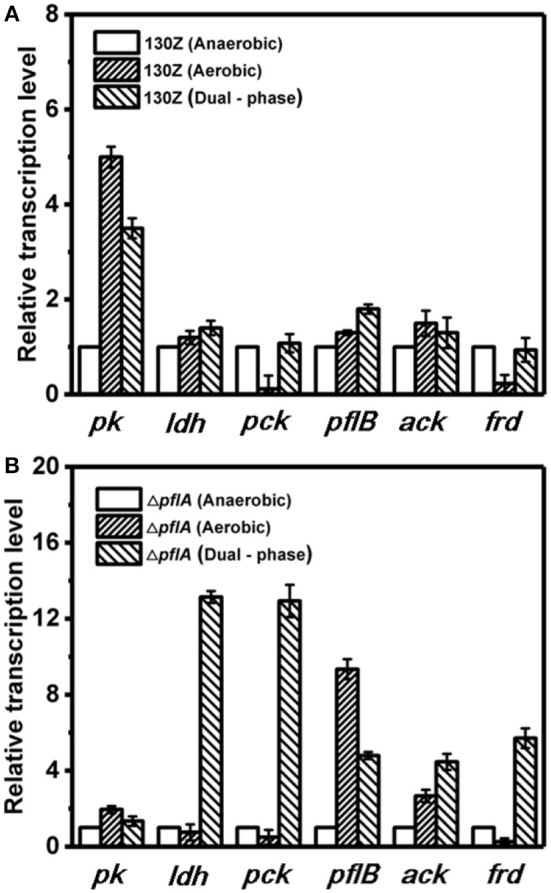
Expression levels of genes related to organic acid biosynthesis pathway in the 130Z strain **(A)** and Δ*pflA* strain **(B)** under different cultivation modes.

Next, mRNA levels of key genes in the same strain under different conditions were also compared. As shown in Figure 6A, wild-type *A. succinogenes* showed the same transcription levels of *ldh* and *ack* among three kinds of cultivation modes. The expression levels of *pck* and *frd* genes were significantly down-regulated under aerobic conditions, in accordance with low carbon flux of SA-producing reductive C4 pathway. A similar phenomenon was observed in the Δ*pflA* strain. Specifically, the expression levels of *ldh, pck*, and *frd* genes under dual-phase conditions were much higher than those under other fermentation modes in the Δ*pflA* strain, leading to massive lactic acid and succinic acid accumulation (Figure 6B). However, although the expression level of *ack* was up-regulated in the Δ*pflA* strain under both aerobic and dual-phase cultivation, higher acetic acid accumulation was observed in the aerobic condition. Instead, no difference in acetic acid production was found between anaerobic and dual-phase cultivation conditions (Figures 3D, 5D). Thus, it was speculated that an alternative route to acetic acid biosynthesis exists in *A. succinogenes*.

## Conclusions

**Figure 7:**
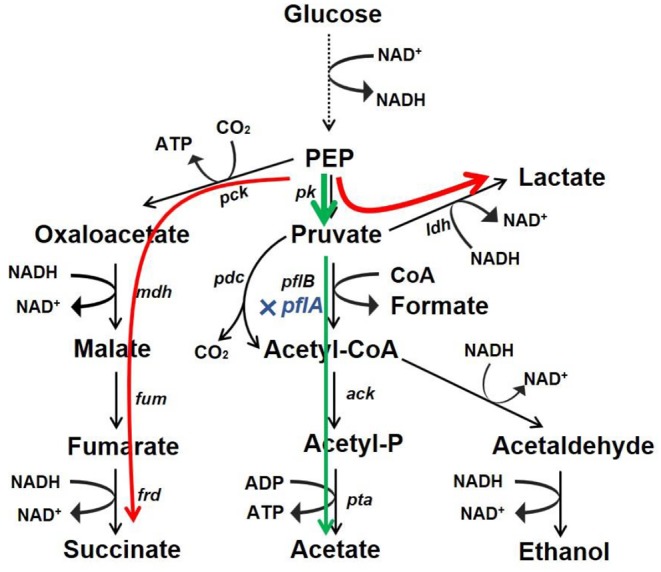
Redistribution of metabolic products by mutant with *pflA* deletion. Glucose carbon flux in strain Δ*pflA* under aerobic condition (green) and anaerobic or dual-phase condition (red).

Microbial production of organic acid is a promising approach owing to advantages of being low cost, efficient, and environment friendly. In this work, we developed a markerless knockout method for *A. succinogenes*. It was found that C3 metabolic pathways were regulated by *pflA*, which was vital for organic acid synthesis. Moreover, the resulting Δ*pflA* strain was able to accumulate acetic acid under aerobic conditions and lactic acid under dual-phase fermentation (Figure 7). These results reveal a potential finely tuned mechanism in organic acid biosynthesis. Moreover, the study indicates that *A. succinogenes* is not only a promising succinate producer, but also potentially an excellent strain for the bioproduction of acetic acid and lactic acid.

## Data Availability

All datasets generated for this study are included in the manuscript/Supplementary Files.

## Author Contributions

WZ designed and performed the experiments, analyzed the results, and wrote the manuscript. QY, MW, and HL assisted in experimental work and analytical work. JZ, WD, and JM assisted in the design of the study. MJ and FX revised the manuscript. All authors read and approved the final manuscript.

### Conflict of Interest Statement

The authors declare that the research was conducted in the absence of any commercial or financial relationships that could be construed as a potential conflict of interest.
