# Supplementary material for: Metabolic Regulation of Organic Acid Biosynthesis in Actinobacillus succinogenes
Source: Front Bioeng Biotechnol. 2019 Sep 18;7:216. doi: 10.3389/fbioe.2019.00216 (PMC6759810; doi:10.3389/fbioe.2019.00216)
Supplement: Supplementary file 1 [file Table_1.DOCX]

Supplementary Table 1. Primers used in this study.

| Genes | Primers | Sequences |
| --- | --- | --- |
| *pflA* | F_0_ | ATGCCGTTTATTCTACCG |
|  | R_0_ | ACAAGCGTGGCAGCAAGGAC |
|  | up-fwd | GAGCTCTTTGATACCGCTATTGCTCC |
|  | up-rev | ATACCGCCCATTTGTGAGCGCAATACAG  ACAACGCATCAAA |
|  | down-fwd | TTTGATGCGTTGTCTGTATTGCGCTC  ACAAATGGGCGGTAT |
|  | down-rev | GAGCTCCGGCGTAGCGATTGTTAT |
| *pk* | fwd | CGTATGAACTTCTCCCACGGAAC |
|  | rev | AAACCCGGATTTTAGGGCCTTG |
| *ldh* | fwd | CGAGGCTTATCGTAACGGCTATC |
|  | rev | CCGTATTGGCGGCTTGATTAATC |
| *pck* | fwd | GAAACCAAACCGGGTTTGGAG |
|  | rev | CACCAAACGGTGTCTTTCGTAG |
| *pfl* | fwd | CGTACGAAGGCGACGAATCATTC |
|  | rev | TGGGAAGTGATGGTTGACGGAG |
| *ack* | fwd | TTCCGGAAGCGCGTATCAAATG |
|  | rev | CGGTGACCGATTGCTACGATAC |
| *frd* | fwd | ATTGCAGCAGCAGAAGCGAAC |
|  | rev | CAACGTCTTGTTCGCACAACCAG |
| *16s* | fwd | GGTGAGTAATGCTTGGGGATCTG |
|  | rev | AGGCCTTTACCCCACCAACTAC |
